# Resolving a Complex Neonatal Phenotype by Rapid Trio Whole‐Genome Sequencing: A De Novo 11q14.3–q22.3 Deletion and a Splicing‐Altering Synonymous *ANK1* Variant

**DOI:** 10.1002/jcla.70289

**Published:** 2026-06-18

**Authors:** Hyun‐Woo Lee, Ja‐Hyun Jang, Beom Hee Lee, Hee‐Jeong Youk, Eun Sun Kim, Kee Hyun Cho, Yun Sil Chang, Heui Seung Jo, Mi‐Ae Jang

**Affiliations:** ^1^ Department of Laboratory Medicine and Genetics, Samsung Medical Center Sungkyunkwan University School of Medicine Seoul Korea; ^2^ Department of Pediatrics, Asan Medical Center University of Ulsan College of Medicine Seoul Korea; ^3^ Department of Laboratory Medicine, Kangwon National University Hospital Kangwon National University School of Medicine Chuncheon Korea; ^4^ Department of Pediatrics, Kangwon National University Hospital Kangwon National University School of Medicine Chuncheon Korea; ^5^ Department of Pediatrics, Samsung Medical Center Sungkyunkwan University School of Medicine Seoul Korea

**Keywords:** genetic counseling, genomics, infant, intrahepatic cholestasis, precision medicine, rapid diagnostic tests

## Abstract

**Background:**

Neonates with complex and evolving phenotypes often lack sufficiently specific clinical features to guide targeted genetic testing. Rapid trio whole‐genome sequencing (WGS) may provide comprehensive etiologic clarification by simultaneously detecting sequence and structural variants.

**Methods:**

Rapid trio WGS was performed in a preterm infant with dysmorphic features, cardiac anomalies, hemolytic anemia, and persistent intrahepatic cholestasis, together with her parents. A copy number variant was confirmed by chromosomal microarray analysis. The functional effect of a candidate single nucleotide variant was investigated using SpliceAI, reverse transcription PCR, agarose gel electrophoresis, and Sanger sequencing.

**Results:**

The genetic diagnosis was achieved within 10 days. WGS identified a de novo heterozygous 15.6 Mb deletion at chromosome 11q14.3–q22.3, which was classified as pathogenic and accounted for the infant's congenital anomalies. A novel paternally inherited *ANK1* synonymous variant, NM_000037.4:c.4104G>A was also identified, and its RNA analysis demonstrated exon 33 skipping, resulting in an in‐frame deletion. Although the variant was classified as a variant of uncertain significance, its demonstrated splicing effect and the concordant spherocytosis phenotype in the infant and her father supported its clinical relevance. This integrated diagnosis informed surveillance, genetic counseling, and evaluation of the persistent cholestasis while avoiding further invasive investigation.

**Conclusion:**

Rapid trio WGS, complemented by functional RNA analysis, resolved distinct components of a complex neonatal phenotype by identifying both a pathogenic chromosomal deletion and a splicing‐altering synonymous *ANK1* variant. This case demonstrates the value of comprehensive genomic testing for precision diagnosis and individualized management in neonatal medicine.

## Graphical abstract

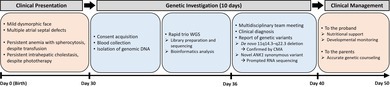

## 1 Introduction

Continued technical advances in next‐generation sequencing have established whole‐genome sequencing (WGS) as a powerful diagnostic tool for rare genetic disorders, particularly in neonates and infants [1, 2]. During the neonatal period, clinical manifestations are often incomplete or nonspecific, limiting the diagnostic yield of phenotype‐driven targeted tests. At the early stage of life, a comprehensive, hypothesis‐free genomic approach is advantageous because it does not rely on predefined clinical features. WGS fulfills that role by enabling the genome‐wide detection of diverse classes of pathogenic variants—including single‐nucleotide variants, small insertions and deletions, and structural variants such as copy number variants (CNVs)—within a single assay [3]. Consistent with those technical strengths, multiple studies have demonstrated that WGS outperforms conventional approaches such as karyotyping, chromosomal microarray analysis (CMA), and targeted gene panels in diagnostic utility, providing clinically actionable diagnoses in neonatal intensive care settings [4, 5]. Moreover, rapid WGS has been shown to reduce morbidity, mortality, and healthcare costs in critically ill infants by facilitating timely diagnoses and precision clinical management [6, 7].

Here, we report a neonatal case that illustrates the integrative diagnostic value of rapid trio WGS, which identified a de novo interstitial deletion involving chromosome 11q that explained the patient's congenital anomalies. However, this CNV alone could not account for the persistent intrahepatic cholestasis of the neonate. The proband's WGS also revealed a novel synonymous variant in *ANK1*, a gene associated with hereditary spherocytosis type 1 (MIM 182900) in an autosomal dominant inheritance pattern. Although this variant is silent at the coding level, a subsequent RNA analysis demonstrated aberrant splicing, confirming its functional effect.

## 2 Materials and Methods

### 2.1 Study Subjects

A preterm infant with complex medical conditions and her parents were evaluated at Kangwon National University Hospital and Samsung Medical Center. Informed consent for genetic testing and research use of biological data was obtained from the parents. This study was approved by the Institutional Review Board of Samsung Medical Center (2025‐03‐144‐001).

### 2.2 Trio WGS

Between 1 and 3 mL of whole blood from the proband and 6 mL from the parents were collected for WGS. Genomic DNA was extracted from peripheral blood leukocytes and then subjected to WGS. Sequencing libraries were prepared using a TruSeq DNA PCR‐Free kit (Illumina, San Diego, CA, USA), and sequencing was performed on a NovaSeq X system (Illumina). Generated sequence reads were aligned to the GRCh38/hg38 public human reference genome with the application of a masking file, as previously described [8], using BWA‐MEM (version 0.7.17). Duplicate reads were marked using Picard (version 2.20.8; http://broadinstitute.github.io/picard/). Variant calling was performed using the HaplotypeCaller module of the Genome Analysis Toolkit (GATK; version 4.1.8). The mean depth of coverage was 41.6×, with 97.8% of bases covered at a depth of ≥ 20×. Variant annotation and prioritization were performed using EVIDENCE v4.3, an automated variant interpretation system developed by 3billion [9]. Variant interpretation was conducted by integrating the proband's clinical information with genomic data, in accordance with the American College of Medical Genetics and Genomics/Association for Molecular Pathology (ACMG/AMP) guidelines and related expert consensus recommendations [10, 11, 12].

### 2.3 Copy‐Number Variation Analysis

Genomic DNA from the proband was additionally analyzed for CNVs using a CMA performed on the CytoScan Dx platform (Thermo Fisher Scientific, Waltham, MA, USA) according to the manufacturer's instructions. The CMA data were analyzed using Chromosome Analysis Suite Software (ChAS; version 4.5.1) based on GRCh38/hg38. The analytical resolution of the assay was approximately 50 kb for copy number gains and 25 kb for copy number losses. Regions of loss of heterozygosity exceeding 3 Mb and mosaic copy number changes greater than 20% were also detectable. Identified CNVs were interpreted with a focus on those involving protein‐coding genes, particularly those listed in Online Mendelian Inheritance in Man (OMIM). Clinical significance was evaluated by comparison with the following publicly available databases: DECIPHER (https://decipher.sanger.ac.uk/), Database of Genomic Variants (http://dgv.tcag.ca), ClinGen (www.clinicalgenome.org) and ClinVar (https://www.ncbi.nlm.nih.gov/clinvar/). CNVs overlapping known pathogenic regions or dosage‐sensitive genes were considered pathogenic [12].

### 2.4 Reverse Transcription PCR (RT‐PCR) and RNA Sequencing

We used the TRIzol method to extract total RNA from lymphocytes isolated from peripheral blood. The extracted RNA was reverse‐transcribed into cDNA using an Omniscript reverse transcriptase kit (Qiagen, Hilden, Germany). The resulting cDNA was amplified using Platinum II HotStart Taq DNA polymerase (Thermo Fisher Scientific) with custom‐designed primers targeting *ANK1* exons 31–34 (forward primer, targeting exon 30: 5′‐GCCAACTTCACCACCAATGT‐3′; reverse primer, targeting the exon 34 and 35 junction: 5′‐TGAGAGAACCTGGTGTGGAC‐3′). PCR products were resolved by agarose gel electrophoresis to assess the amplicon size relative to the expected transcript. For further validation, PCR products were subjected to Sanger sequencing using an ABI 3730xl DNA analyzer (Applied Biosystems, Foster City, CA, USA). Sequence data were analyzed using Sequencher software (Gene Codes Corp., Ann Arbor, MI, USA) and aligned to the *ANK1* reference transcript (NM_000037.4). Gel images were acquired from samples processed in parallel within the same experimental run to comply with institutional digital image integrity guidelines.

### 2.5 Prediction of Alternative Splicing

The potential splicing effect of a variant was assessed using SpliceAI [13]. Delta scores ≥ 0.2 were considered indicative of a deleterious effect on normal splicing. The affected splice donor or acceptor site and the corresponding delta score are reported.

## 3 Results

### 3.1 Clinical Investigation

The patient was born at 33^+5^ weeks of gestation via cesarean section, with a birth weight of 1450 g (6th percentile for gestational age). Prenatal ultrasonographic examinations revealed fetal growth restriction but showed no major structural abnormalities, hepatosplenomegaly, fetal hydrops, or other abnormal prenatal findings suggestive of a syndromic disorder. At birth, mild dysmorphic facial features were noted, including a narrow and flat forehead, long face, and micrognathia. The infant developed respiratory distress immediately after birth and required positive pressure ventilation. The initial laboratory evaluation revealed a hemoglobin level of 14.7 g/dL and a total bilirubin level of 4.42 mg/dL. At 24 h of life, the total bilirubin level had increased to 14.4 mg/dL, and intensive phototherapy was initiated, resulting in prompt improvement to 8.4 mg/dL. At that time, the direct bilirubin level was 0.96 mg/dL, and aspartate aminotransferase (AST)/alanine aminotransferase (ALT) levels were 57/13 U/L. On day 10 of life, laboratory testing showed a hemoglobin level of 7.5 g/dL, reticulocyte count of 10.3%, prothrombin time international normalized ratio of 1.10, activated partial thromboplastin time of 36.2 s, total bilirubin of 9.3 mg/dL, and direct bilirubin of 1.99 mg/dL. Both direct and indirect antiglobulin tests were negative. The patient received one transfusion of packed red blood cells, after which the hemoglobin level stabilized at approximately 10.5 g/dL. At 1 month of age, laboratory studies demonstrated persistent cholestasis, with a total bilirubin level of 5.77 mg/dL, direct bilirubin of 3.3 mg/dL, and AST/ALT levels of 122/110 U/L. Genetic testing was performed to investigate the underlying cause of intrahepatic cholestasis. A peripheral blood smear (PBS) revealed normocytic normochromic anemia with polychromasia, anisocytosis, tear drop cells, elliptocytes, spherocytes, and schistocytes. Brain and abdominal ultrasonography showed no significant abnormalities. Echocardiography demonstrated multiple atrial septal defects with a moderate‐to‐large shunt. The proband's father was 33 years old and previously healthy; however, he exhibited spherocytosis on his PBS, similar to the proband. The proband's mother was also healthy, without relevant medical history, and did not exhibit any abnormal PBS findings. The patient was discharged on day 50 of life after stabilization of respiratory status and feeding. At the most‐recent follow‐up, corresponding to a corrected age of 4 months, all liver function tests have normalized, and anemia is no longer present. Both length and weight are below the 3rd percentile, but head circumference is at the 50th percentile. The infant is able to establish eye contact, but feeding remains prolonged with insufficient intake; therefore, the patient is being followed closely by a nutrition support team.

### 3.2 Genetic Investigation

**FIGURE 1:**
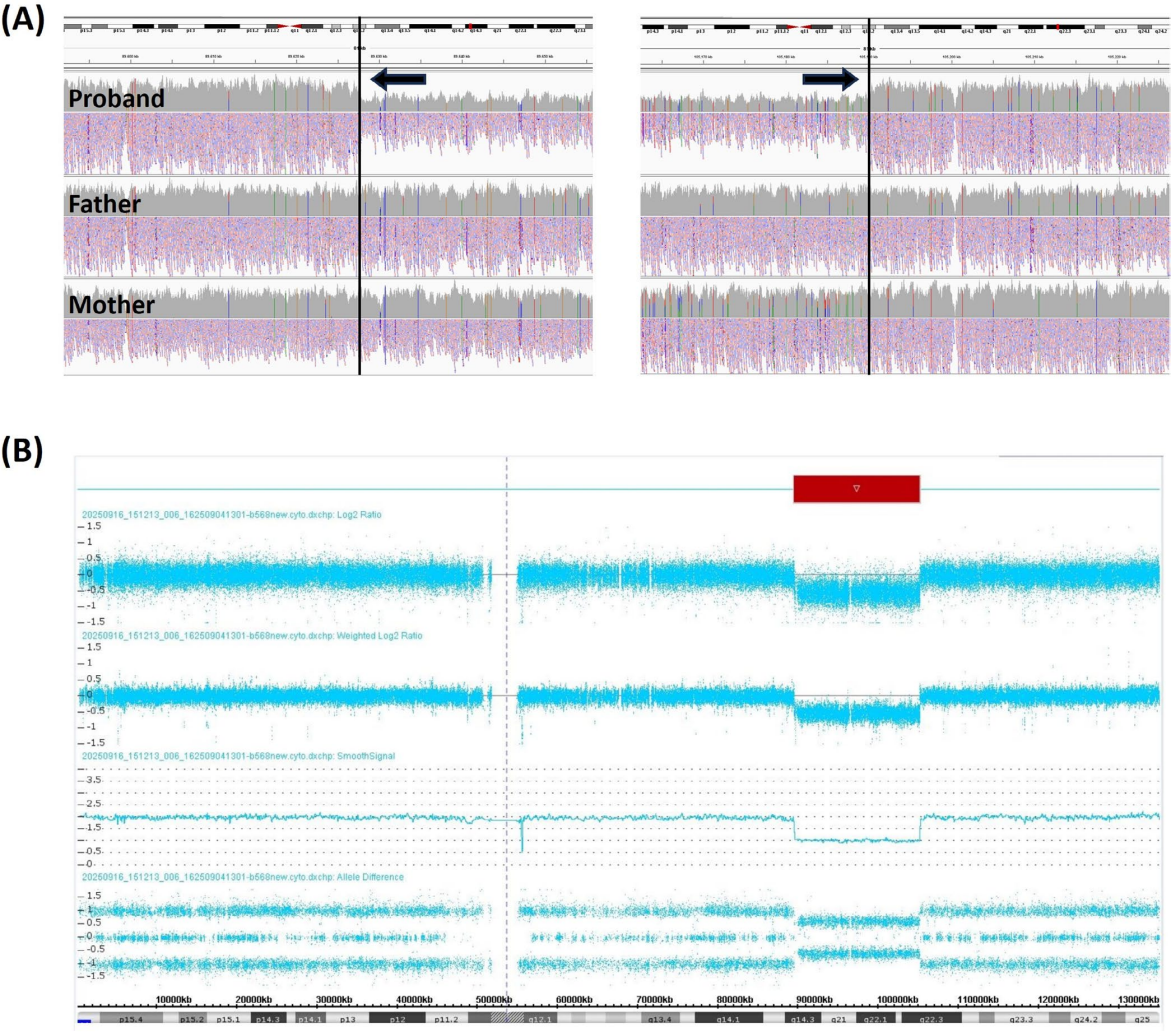
Characterization of a 15.6‐Mb deletion at 11q14.3q22.3 in a neonate with congenital anomalies and hemolytic anemia. (A) Trio WGS detected a de novo heterozygous deletion at chromosome 11q14.3–q22.3 (NC_000011.10:g.89627668_105190097del) in the proband. The black vertical lines and arrows indicate the deletion breakpoints, with marked decreases in read depth observed around both breakpoints in the proband, compared with the parental samples. (B) Chromosomal microarray analysis results from a CytoScan Dx assay demonstrate a log2 ratio and allele difference consistent with the identified 15.6‐Mb deletion at 11q14.3–q22.3. WGS, whole genome sequencing.

Trio WGS was performed on the proband and her parents. WGS of the proband revealed a de novo heterozygous 15.6‐Mb interstitial deletion spanning chromosome 11q14.3–q22.3 (NC_000011.10:g.89627668_105190097del) (Figure 1A). The CNV analysis confirmed the presence of 11q interstitial deletion [arr (GRCh38) 11q14.3q22.3 (89,624,404–105,189,638) × 1] (Figure 1B). The deleted region encompasses 66 genes, including 59 genes listed in OMIM. Among them, six genes—*AMOTL1*, *MMP13*, *MTNR1B*, *PANX1*, *TRPC6*, and *YAP1*—are associated with autosomal dominant inheritance. Of those dominantly inherited genes, *YAP1* exhibited evidence of haploinsufficiency, with a pLI score of 1.0 and a DECIPHER HI index of 9.76%. According to the ACMG technical standards for CNV interpretation, the identified deletion received a total score of 1.20 and was therefore classified as pathogenic (score > 0.99).

**FIGURE 2:**
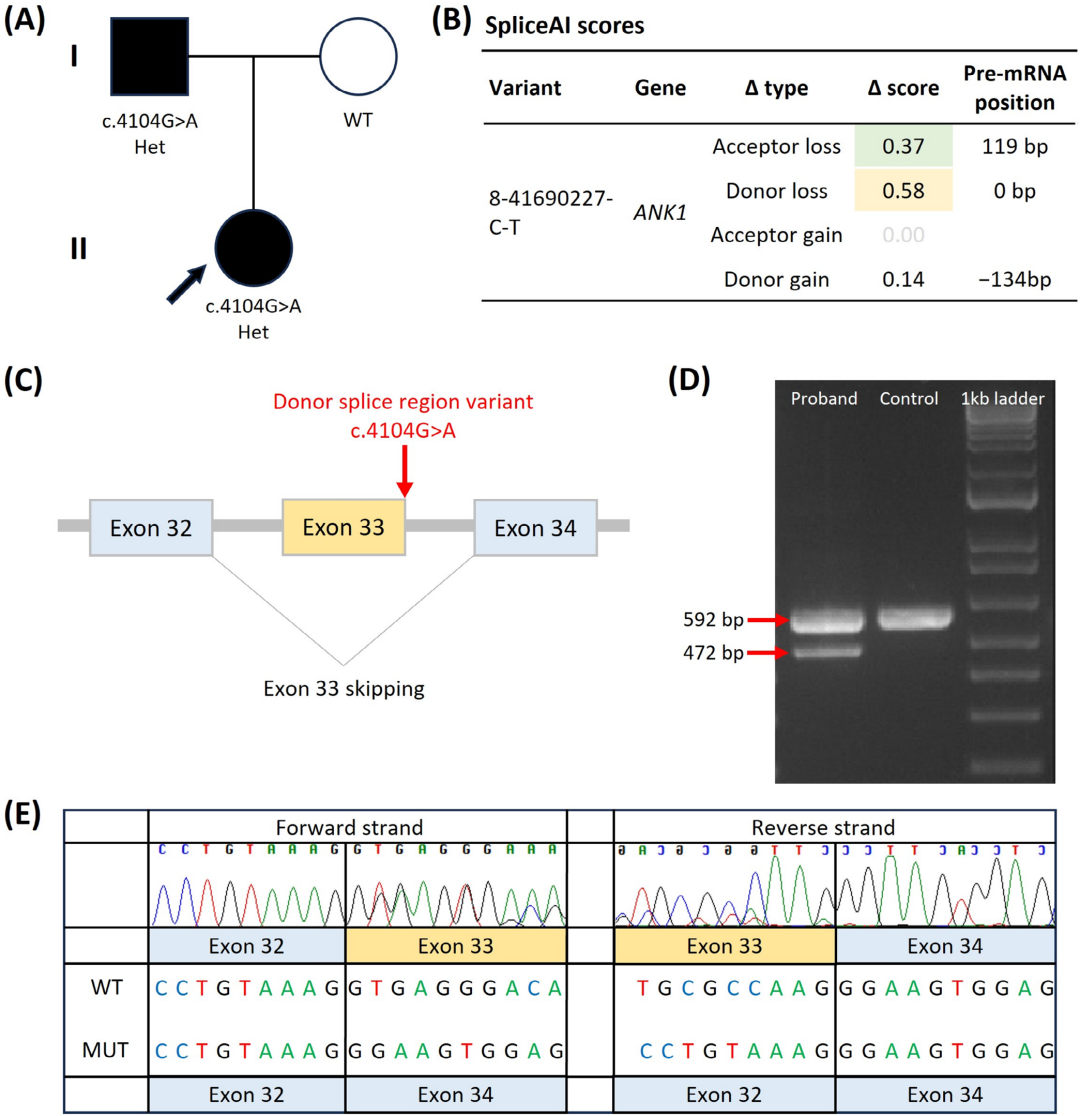
Splicing effect of the novel synonymous *ANK1* variant NM_000037.4:c.4104G>A. (A) Pedigree of the family with hereditary spherocytosis. (B) SpliceAI scores of the *ANK1* variant. (C) Schematic representation of targeted RNA sequencing and the resulting aberrant cDNA transcript. (D) Gel electrophoresis showing an additional band in the proband that was 120 bp shorter (472 bp) than in the control (592 bp). (E) Sanger sequencing confirmed exon 33 skipping (NM_000037.4:r.3985_4104del), resulting in an in‐frame deletion of 40 amino acids. bp, base pair; Het, heterozygous; MUT, mutant; WT, wild type.

In addition, trio WGS identified a novel heterozygous *ANK1* variant, NM_000037.4:c.4104G>A, in the proband and her father, but not in her mother (Figure 2A). This variant is synonymous and does not alter the amino acid sequence. However, an *in silico* splicing analysis using SpliceAI predicted the loss of a donor splice site at position c.4104, with a high delta score of 0.58, suggesting exon 33 skipping (Figure 2B,C). RT‐PCR followed by agarose gel electrophoresis demonstrated that wild‐type cDNA produced a single band of the expected size (592 bp), whereas the proband cDNA produced an additional band approximately 120 bp shorter than the wild‐type PCR product (Figure 2D). Subsequent Sanger sequencing confirmed the presence of an aberrant transcript (472 bp), consistent with exon 33 skipping, as predicted by SpliceAI (Figure 2E). This splicing alteration resulted in an in‐frame deletion of 120 bp, r.3985_4104del, corresponding to the loss of 40 amino acids, p.(Val1329_Lys1368del), without disruption of the reading frame. According to the ACMG/AMP guidelines, the PVS1_moderate criterion was applied instead of PP3, based on the results of the splicing analysis. This variant also met the PM2 criterion because it was absent from population databases. Taken together, the *ANK1* synonymous variant c.4104G>A was classified as a variant of uncertain significance (VUS), despite its demonstrated effect on mRNA splicing.

## 4 Discussion

**FIGURE 3:**
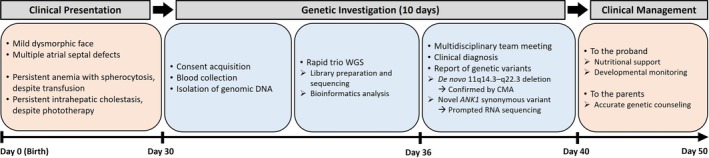
Clinical utility of trio WGS and time metrics in a neonate with congenital anomalies and hemolytic anemia. CMA, chromosomal microarray; WGS, whole genome sequencing.

This case highlights the clinical utility of a rapid trio WGS protocol in the neonatal setting. From blood sampling to a definitive genetic diagnosis, only 10 days were required, and multidisciplinary interpretation involving clinical geneticists enabled the identification of clinically meaningful variants (Figure 3). Notably, the only antenatal finding was isolated fetal growth restriction, without specific structural abnormalities suggestive of an underlying syndromic disorder, emphasizing the diagnostic value of rapid trio WGS even in neonates with limited prenatal clues. The de novo 11q14.3–q22.3 deletion explained the patient's dysmorphic features and cardiac defects [14], and a novel *ANK1* variant accounted for the persistent hyperbilirubinemia and was shown to be paternally inherited. These findings not only clarified a complex phenotype but also allowed anticipatory management and accurate genetic counseling, with a 50% recurrence risk for the *ANK1* variant and a low recurrence risk for the de novo 11q deletion in subsequent offspring.

To date, eight cases with overlapping deletions involving 11q14.3–q22.3 have been reported; six were classified as pathogenic [14, 15, 16, 17, 18, 19], and the other two were interpreted as variants of uncertain pathogenicity [20, 21]. Review of those cases indicates that impaired growth and developmental delay are the most consistently observed features. Based on that information, the early involvement of a nutrition support team and careful longitudinal developmental monitoring were initiated in the present patient. Phenotypic differences among previously reported cases are thought to result from differences in breakpoint locations and the specific gene content within the deleted regions. In this patient, only one dominantly inherited gene within the deleted segment showed clear evidence of haploinsufficiency: *YAP1*. According to OMIM, heterozygous loss‐of‐function mutations in *YAP1* are associated with ocular coloboma, hearing impairment, cleft lip/palate, and impaired intellectual development. Those phenotypes were observed in 13 individuals with *YAP1* haploinsufficiency reported previously (ages 5–57) [22]. Although those manifestations are not yet evident due to the patient's young age, the presence of *YAP1* deletion provides a clear rationale for additional ophthalmologic and otolaryngologic surveillance. Ten autosomal recessive genes were also included in the deleted region—*CEP295*, *MED17*, *HEPHL1*, *MRE11*, *CEP57*, *MTMR2*, *PGR*, *CFAP300*, *MMP20*, and *DYNC2H1*. No pathogenic variant (PV), likely pathogenic variant (LPV), or VUS was identified in the corresponding genes on the homologous chromosome 11, suggesting that these recessive genes are unlikely to contribute to the phenotype.

In neonates with persistent intrahepatic cholestasis, biliary atresia and a broad range of genetic disorders, such as Alagille syndrome, neonatal intrahepatic cholestasis caused by citrin deficiency, and Dubin‐Johnson syndrome, should be considered in the differential diagnosis [23]. Given that hereditary spherocytosis typically presents with unconjugated hyperbilirubinemia and presentation with intrahepatic cholestasis is rarely reported [24], the possibility of hereditary spherocytosis was not initially considered, even by pediatric hepatologists. However, the early genetic diagnosis using rapid trio WGS, combined with a multidisciplinary evaluation, enabled a comprehensive interpretation of the patient's condition and led to the conclusion that the novel *ANK1* variant could be responsible for the intrahepatic cholestasis. As a result, further investigations for persistent jaundice, including invasive procedures such as liver biopsy, were avoided, and appropriate prognostic assessment and follow‐up were established. Although the *ANK1* variant identified in this patient was initially classified as a VUS, the concordant spherocytosis phenotype observed in both the proband and her father strongly supported its disease relevance. As additional case reports accumulate and genotype–phenotype correlations and co‐segregation are established, further evidence codes such as PS4 and PP1 might be applied, allowing potential reclassification of this variant to likely pathogenic or pathogenic. Furthermore, although not identical to the variant identified in this patient, two variants affecting the same splice region have previously been reported. The first variant, NM_000037.4(*ANK1*):c.4104G>C, was submitted to ClinVar as likely pathogenic. The second variant, NM_000037.4(*ANK1*):c.4104+1G>T, was reported in Human Gene Mutation Database as a disease‐modifying variant and interpreted as pathogenic based on the PVS1_S, PS2, and PM2_P criteria [25]. Taken together, despite being a synonymous change, the *ANK1* variant identified in this patient has been experimentally shown to cause exon 33 skipping, exhibits phenotypic correlation, and is supported by prior reports of LPV/PV at the same splice site, warranting careful attention in future literature.

Several limitations should be acknowledged. First, this report represents a single case, and genotype–phenotype correlations for the novel *ANK1* variant require validation through additional reports. Second, although aberrant splicing was demonstrated through RNA studies, quantitative analyses of transcript levels and protein functions were not performed. Third, the long‐term clinical manifestations associated with *YAP1* haploinsufficiency cannot yet be determined due to the patient's young age and will require longitudinal observation. Fourth, although rapid trio WGS proved highly informative in this case, implementation of such testing could be influenced by institutional resources, cost considerations, and the availability of multidisciplinary expertise.

In conclusion, this case demonstrates how rapid trio WGS can provide comprehensive etiologic clarification in neonates with complex and evolving clinical features. The identification of both a pathogenic 11q14.3–q22.3 deletion and a functionally significant synonymous variant within a single assay allowed precise explanations of distinct aspects of the patient's phenotype. Beyond diagnosis, the genomic findings directly influenced clinical management, surveillance strategies, and genetic counseling. These results underscore the expanding role of rapid WGS as a powerful tool for precision diagnosis and individualized care in neonatal medicine.

## Author Contributions

**Hyun‐Woo Lee:** conceptualization, data curation, visualization, writing – original draft. **Ja‐Hyun Jang, Beom Hee Lee, Hee‐Jeong Youk, Eun Sun Kim, Kee Hyun Cho:** data curation, investigation, writing – review and editing. **Yun Sil Chang:** conceptualization, resources, writing – review and editing. **Heui Seung Jo:** conceptualization, investigation, project administration, supervision, writing – review and editing. **Mi‐Ae Jang:** conceptualization, data curation, investigation, methodology, project administration, supervision, writing – review and editing.

## Funding

This research was supported by the National Institute of Health (NIH) under research project No. 2025‐ER0705‐00.

## Ethics Statement

This study was approved by the Institutional Review Board of Samsung Medical Center (2025‐03‐144‐001). The studies were conducted in accordance with all local legislation and institutional requirements. Written informed consent for participation in this study was provided by the legal guardians of the participants.

## Conflicts of Interest

The authors declare no conflicts of interest.

## Data Availability

Data will be made available on request.
